# Analysis of the correlation between RFC4 expression and tumor immune microenvironment and prognosis in patients with cervical cancer

**DOI:** 10.3389/fgene.2025.1514383

**Published:** 2025-05-19

**Authors:** Bifen Huang, Jianqing Zheng, Bizhen Chen, Min Wu, Lihua Xiao

**Affiliations:** ^1^ Department of Obstetrics and Gynecology, People’s Hospital Affiliated of Quanzhou Medical College, Quanzhou, Fujian, China; ^2^ Department of Radiation Oncology, The Second Affiliated Hospital of Fujian Medical University, Quanzhou, Fujian, China

**Keywords:** cervical cancer, replication factor C subunit 4 (RFC4), tumor immune microenvironment, immune-related genes, immunochemistry, pan-cancer analysis

## Abstract

**Background:**

Replication factor C subunit 4 (RFC4) plays a critical role in the initiation and progression of some cancers; however, its relationship with tumor-infiltrating immune cells in cervical cancer (CC) has not been comprehensively analyzed. This study aimed to determine whether RFC4 overexpression affects overall survival in CC and to explore its impact and potential mechanisms on the tumor immune microenvironment.

**Methods:**

Data from Genotype-Tissue Expression database (GTEx) and Cancer Genome Atlas (TCGA) database were used as the exploration set. Datasets from the Gene Expression Omnibus (GEO) were used as the validation set. We also validated the expression of the RFC4 protein in the Human Protein Atlas (HPA) database and a real cohort. Clinical data on CC were evaluated for their association with RFC4 using TCGA and GEO databases. Possible relationships amongst RFC4, immune cells, and related genes were investigated using Cell-type Identification by Estimating Relative Subsets of RNA Transcripts (CIBERSORT) and Estimation of STromal and Immune cells in MAlignant Tumor tissues using Expression (ESTIMATE). GO and KEGG pathway enrichment analyses were used to explore potential mechanisms. Tumor immune dysfunction and exclusion (TIDE) scores were used to predict the immunotherapeutic response to RFC4.

**Results:**

In the exploration, validation, and real cohort datasets, RFC4 expression was significantly elevated in CC tissues compared to that in normal tissues. Survival analysis based on TCGA and GEO datasets showed that CC patients with high RFC4 expression had a better prognosis than those with low expression. RFC4 expression was strongly correlated with some immunostimulators and immunoinhibitors. RFC4 expression was significantly negatively correlated with activated mast cell immune infiltration, activated CD4 memory T cells, M0 macrophages, and resting natural killer (NK) cells and significantly positively associated with activated dendritic cells, resting dendritic cells, and plasma cells.

**Conclusion:**

RFC4 is highly expressed in CC tissues. However, patients with high RFC4 expression in CC have a better prognosis, possibly because RFC4 exerts antitumor effects by affecting the immunostimulatory tumor microenvironment, such as immunostimulatory and dendritic cell infiltration.

## 1 Introduction

Cervical cancer (CC) is one of the most common gynecological malignancies and life-threatening health issue worldwide. Its incidence rate and mortality remain high, especially in developing countries (Siegel et al., 2021; Buskwofie et al., 2020; Bray et al., 2024; Arbyn et al., 2020; Zhang et al., 2020). Despite significant progress in screening and prevention measures for CC in recent years, a deep understanding of the molecular mechanisms underlying CC remains key to improving treatment efficacy and patient survival (Balasubramaniam et al., 2019).

In recent years, significant progress has been made in the treatment of CC, resulting in a significant reduction in patient mortality (Sharma et al., 2020). The implementation of targeted therapies has benefited patients with advanced CC, as these treatments have been effective and significantly reduce the mortality rate of advanced CC (Liontos et al., 2019). In addition to targeted treatment, the use of immune checkpoint inhibitors (ICIs) has greatly improved CC prognosis (Ferrall et al., 2021). However, the overall effectiveness of ICIs is poor, and depends on the sensitivity and expression of driver genes in cancer tissues (Volkova et al., 2021). It is important to understand the genetic mechanism of immunotherapy resistance, which is currently a cutting-edge oncology topic attracting the interest of many scholars.

Human replication factor C (RFC) is a multimeric protein composed of five distinct subunits that have been highly conserved throughout evolution (Yao and O'Donnell, 2012). The function of the RFC family (RFCs) is to load proliferating cell nuclear antigen (PCNA) onto DNA as ATP dependent clamp loaders during DNA synthesis (Chen et al., 2021). In addition, the RFC family plays an important role in DNA repair after DNA damage. The enhanced activity of RFC family members is also an important characteristic of cancer occurrence and development. Among the RFCs, the RFC4 gene encodes the fourth subunit of the RFC complex and is involved in DNA replication as a clamp loader. RFC4 is exhibited highly expressed and has significantly increased activity in various malignant tumors, including prostate, cervical, and colorectal cancers (Krause et al., 2001; Kang et al., 2009; Misbah et al., 2024). However, the role of RFC4 in CC initiation and progression remains unclear. In this study, we investigated the expression of RFC4 in CC and determined its potential biological function and impact on CC prognosis and immunoregulation.

## 2 Materials and methods

### 2.1 Study protocol

**FIGURE 1:**
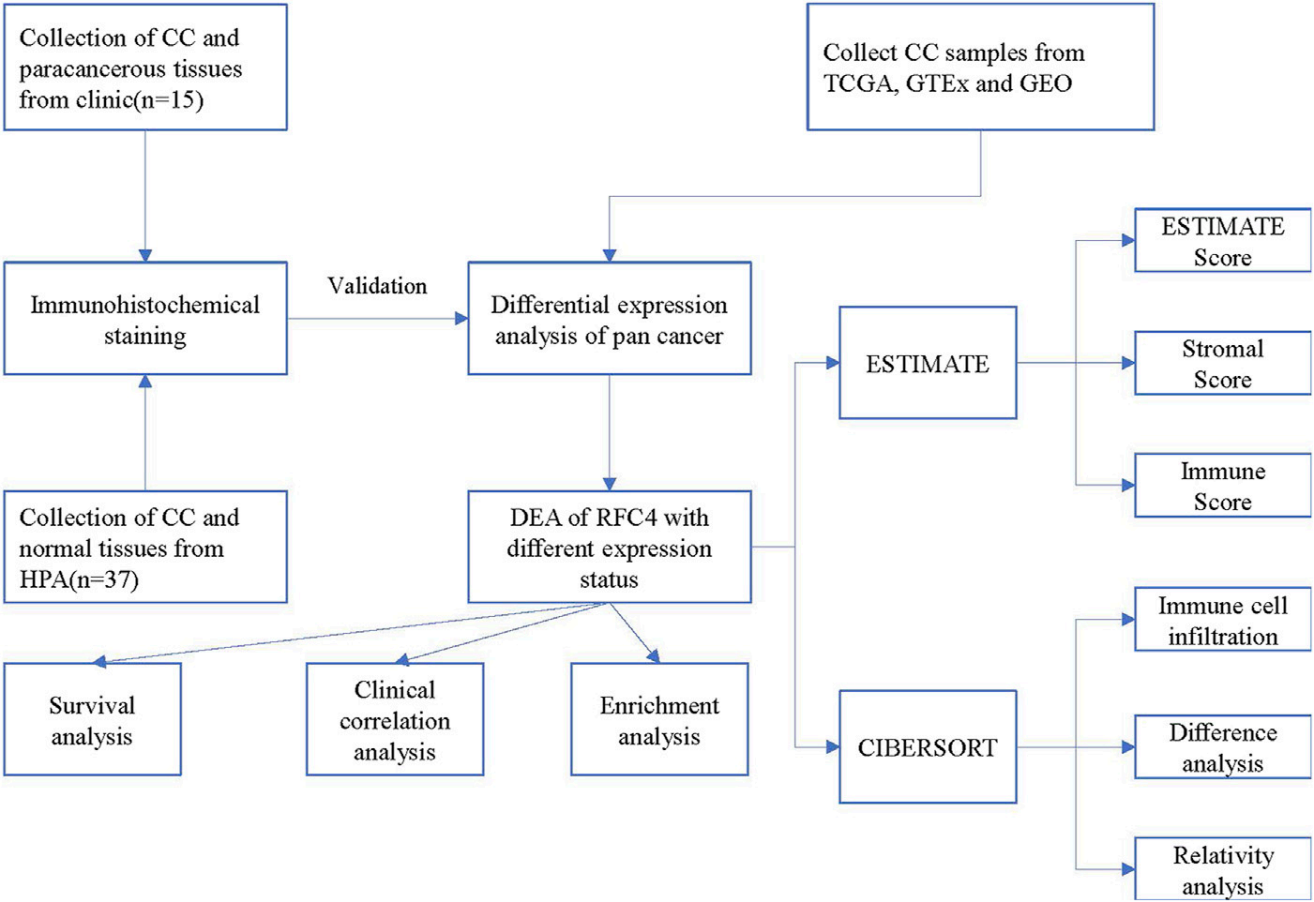
Study design flowchart.

The current study consisted of two parts: one explored the different expression of RFC4 and determined its potential biological function and impact on CC prognosis and immunoregulation using bioinformatics analysis, and the other explored different levels of RFC4 expression using immunohistochemistry (IHC) of 15 patients with CC recruited from the Second Affiliated Hospital of Fujian Medical University. This study was approved by the ethics committee of the Second Affiliated Hospital of Fujian Medical University (2020-06). The study flowchart is shown in Figure 1.

### 2.2 Analysis of RFC4 mRNA and protein expression in CC tissues and adjacent tissues

Pan-cancer expression data were downloaded from Genotype-Tissue Expression database (GTEx; https://www.gtexportal.org/home/) and Cancer Genome Atlas (TCGA; https://www.cancer.gov/ccg/research/genome-sequencing/tcga) databases in May 2024. The RFC4 expression was examined using GTEx and TCGA datasets and verified using Gene Expression Omnibus (GEO; https://www.ncbi.nlm.nih.gov/geo/) and Human Protein Atlas (HPA; https://www.proteinatlas.org/). Differential expression analysis of RFC4 mRNA was conducted using the TCGA dataset for preliminary analysis, and the GTEx and GEO datasets for validation. RFC4 and CC-associated genes were identified using log fold change (|log2FC|) values >1 and *P* < 0.05.

### 2.3 Relationship between RFC4 and clinicopathological characteristics and prognosis of CC patients

The clinicopathological data for survival analysis were identified using the TCGA and GEO (GSE44001) datasets. The relationship between RFC4 mRNA and different clinicopathological characteristics, such as age, pathological type, and clinical stage, was explored using data from the TCGA database. The *surv_cutpoint* function in the *survminer* package was used to determine the best cutoff value of RFC4 mRNA expression in the samples. Using the best cutoff value, patients with CC were divided into high-expression (RFC4^High^) and low-expression (RFC4^Low^) groups. The Kaplan-Meier method was used to plot the overall survival (OS) curves of the two groups, and the log rank test was used for comparisons. The relationship between the RFC4 expression and OS was verified using the GSE44001 dataset. Differences were considered statistically significant at *P* < 0.05.

### 2.4 Screening and functional enrichment analysis of RFC4-related differentially expressed genes (DEGs) in CC

We used the “limma” package to identify DEGs in CC tissues, and then correlation analysis was used to identify RFC4-associated DEGs. The correlation between different gene expression levels was analyzed using Pearson’s correlation coefficient, with *P* < 0.05 indicating statistical significance. The top 50 common DEGs closest to RFC4 were selected, and a heatmap was plotted. RFC4-related DEGs with Pearson correlation coefficient >0.5 and *P* < 0.05 were selected to enrichment analysis. Gene ontology (GO) functional enrichment analysis of the cell composition, biological processes, and molecular function and Kyoto Encyclopedia of Genes and Genomes (KEGG) were conducted for pathway enrichment analysis.

### 2.5 Analysis of the relationship between RFC4 and tumor immune cell infiltration and immunoregulatory factors

Using the *Spearman* correlation analysis, the correlation between RFC4 expression and various infiltrating immune cells in the tumor microenvironment was explored and analyzed, with P < 0.05 considered statistically significant. The immune regulatory factors gene set was selected from previously reported references (Zhang et al., 2021; Gao et al., 2023; Li et al., 2020). The correlation analysis between immunoregulatory factors and RFC4 expression was shown using lollipop plots.

We used Cell-type Identification By Estimating Relative Subsets Of RNA Transcripts (CIBERSORT) to analyze the infiltration of different immune cells in different RFC4-epxressing tumor tissues (Chen et al., 2018). In addition, Estimation of STromal and Immune cells in MAlignant Tumor tissues using Expression (ESTIMATE) data were used to predict the tumor purity and stromal/immune cell infiltration in tumor tissues with different RFC4 expression levels (Yoshihara et al., 2013).

### 2.6 RFC4 protein expression in CC

Data of RFC4 protein expression in CC and normal tissues were retrieved from the Human Protein Atlas database (HPA; http://www.proteinatlas.org/). IHC staining for RFC4 protein were conducted using tissues from 15 patients with CC who were recruited from the Second Affiliated Hospital of Fujian Medical University. All paraffin-embedded tissues originated from donations from surgical patients, and written informed consent was obtained from each donor.

## 3 Results

### 3.1 RFC4 expression in pan-cancer and CC tissues

**FIGURE 2:**
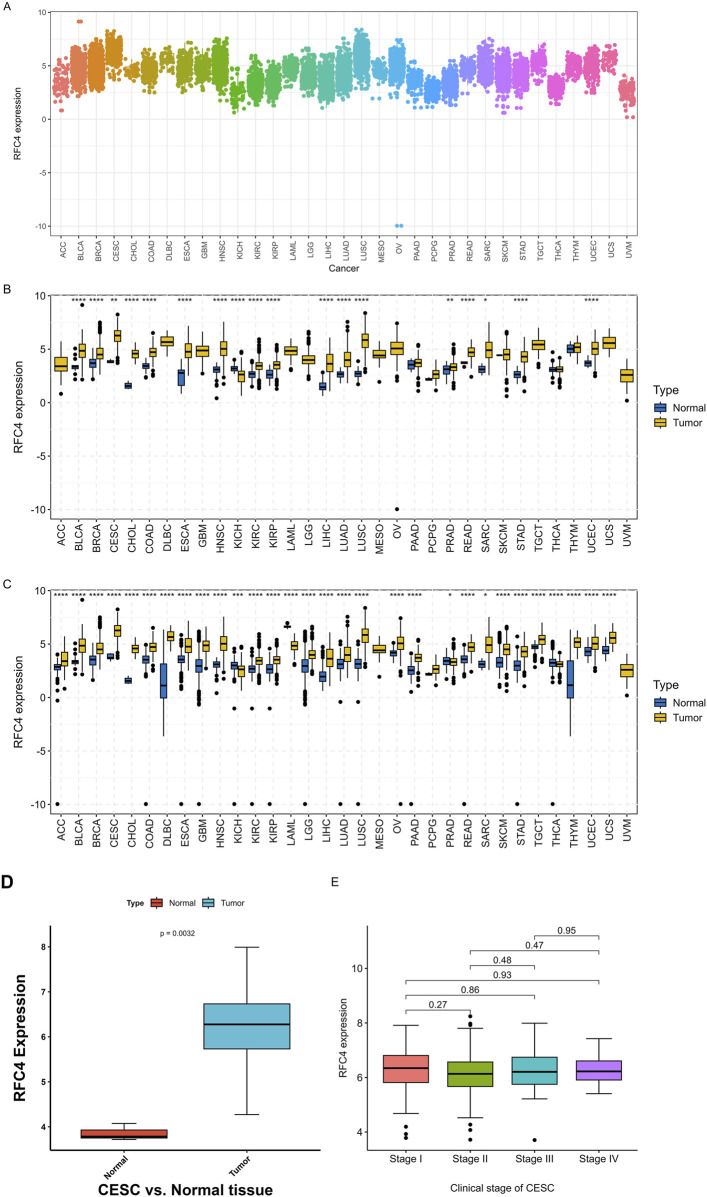
RFC4 expression levels between human cancer and normal tissues. **(A)** Pan-cancer analysis of RFC4 expression across all tumor samples. **(B)** Pan-cancer analysis of RFC4 expression between human cancer and normal tissues from TCGA and GTEx database. **(C)** Validation of pan-cancer analysis of RFC4 expression between human cancer and normal tissues from TCGA and GTEx database. **(D)** The RFC4 expression levels between tumor and normal tissues in CESC from TCGA. **(E)** The RFC4 expression levels among different clinical stage in CESC from TCGA. (ns, nonsignificant; *P* > 0.05; *, *P* < 0.05; **, *P* < 0.01; ***, *P* < 0.001; ****, *P* < 0.0001).

**FIGURE 3:**
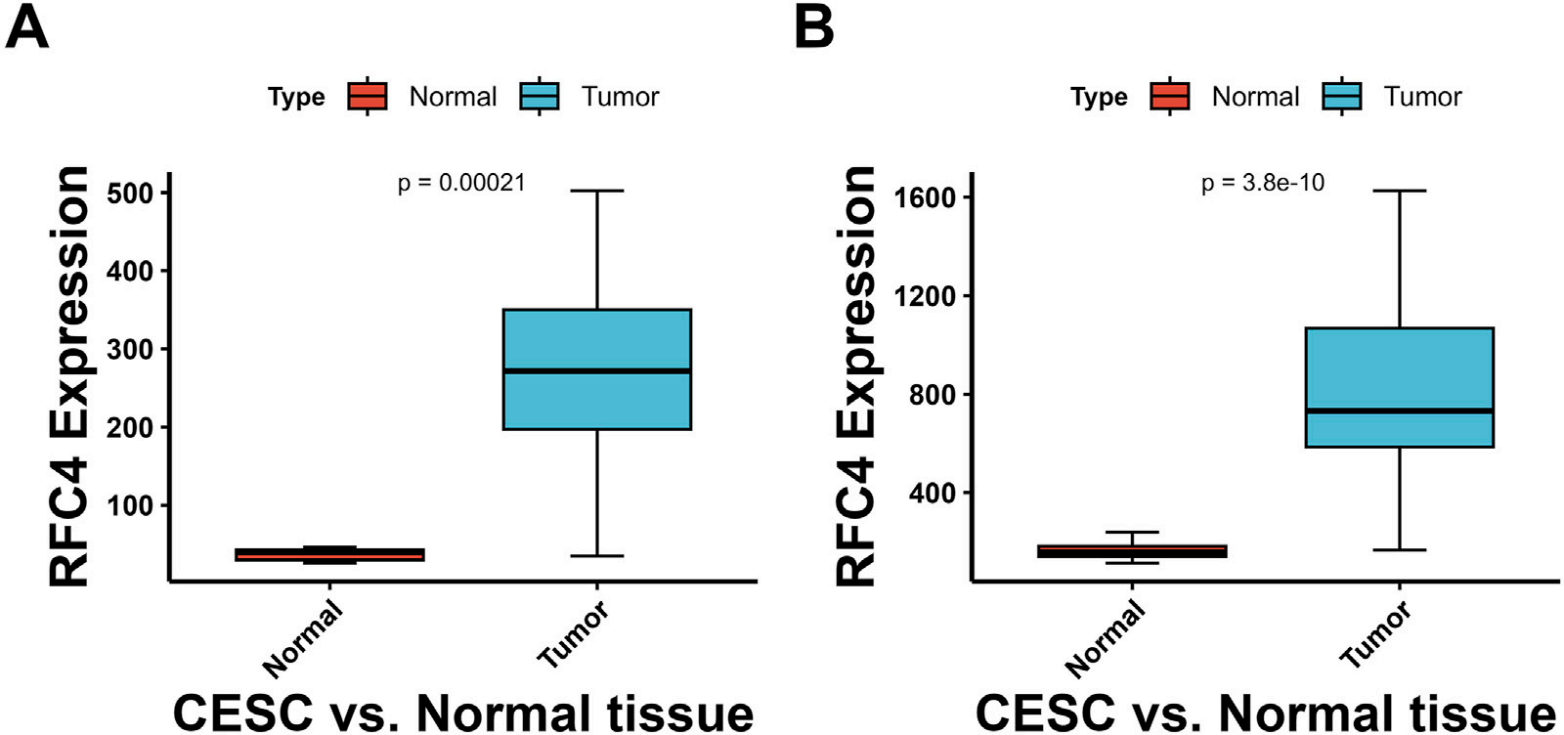
Validation of RFC4 expression levels between CESC and non-cancer tissues form GEO database. Results from the **(A)** GSE39001 and **(B)** GSE67522 datasets.

The differential expression of RFC4 between pan-cancer and normal tissues is shown in Figures 2A,B. The expression level of RFC4 was significantly upregulated in most cancer tissues compared to that in normal tissues in TCGA samples. We further expanded the normal tissues from the GTEx database and found that the expression of RFC4 in cancer tissues was still higher than that in normal tissues, as shown in Figure 2C. Using the Wilcoxon non-parametric test, we found that the expression level of RFC4 in CC tissues was significantly increased compared to that in normal tissues (*P* = 0.0031), as shown in Figure 2D. However, we found no statistically significant difference in the expression level of RFC4 among different stages of CC, as shown in Figure 2E. The differential expression of RFC4 in CC was further validated using the GEO dataset GSE39001 and GSE67522, as shown in Figures 3A,B. The expression of RFC4 was also significantly increased in the validation sets.

### 3.2 Relationship between RFC4 and clinical characteristics of patients with CC

**FIGURE 4:**
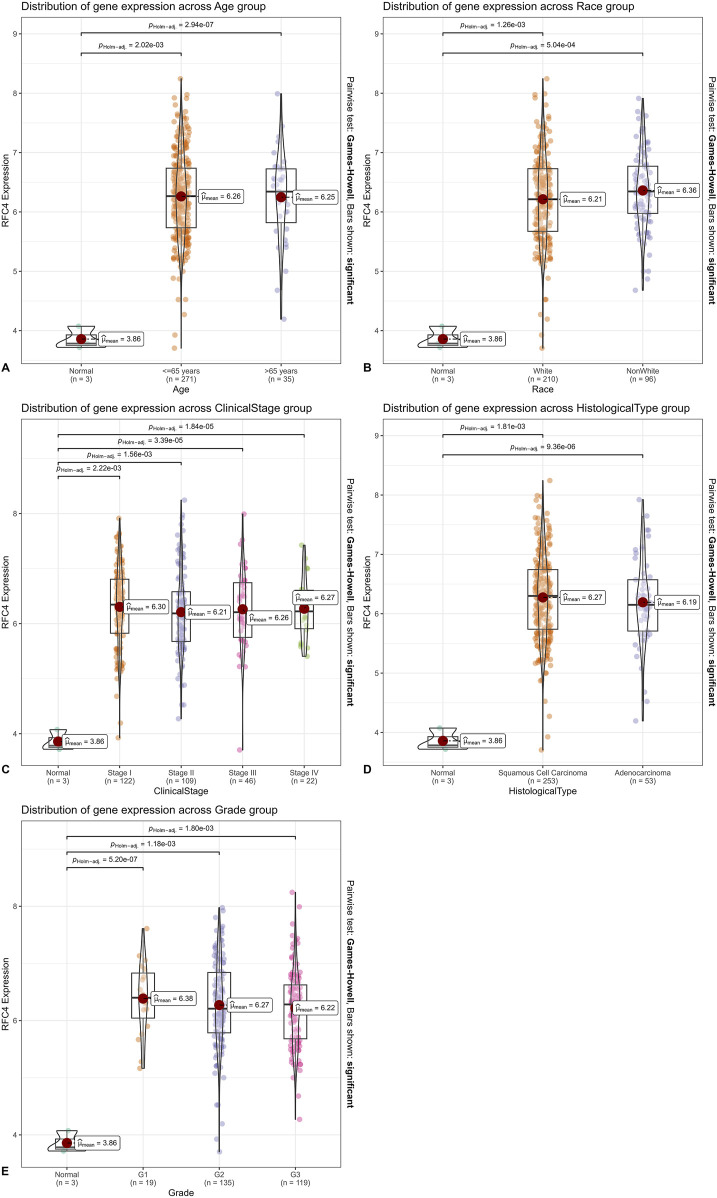
Relationship between RFC4 and clinical characteristics of patients with CC. Classified by **(A)** age. **(B)** Race. **(C)** Clinical stage. **(D)** Histological type. **(E)** Tumor grade.

In the TCGA database, RFC4 mRNA expression levels in CC tissues of patients aged ≤65 and >65 years were significantly higher than those in normal tissues adjacent to the cancer (*P* < 0.001); however, no statistically significant difference was observed between patients aged ≤65 and >65 years (*P* > 0.05) (Figure 4A). Similar results were observed in different races (Figure 4B). The RFC4 mRNA expression levels in CC tissues at clinical stages I, II, III, and IV were significantly higher than those in normal tissues adjacent to the cancer (*P* < 0.001); however, no statistically significant difference was observed among the four stages (*P* > 0.05) (Figure 4C). The RFC4 mRNA expression levels in cervical squamous cell carcinoma, and adenocarcinoma tissues were significantly higher than those in adjacent normal tissues (*P* < 0.001); however, no statistically significant difference was observed between the two pathological types (*P* > 0.05) (Figure 4D). The RFC4 mRNA expression levels in different tumor grades were significantly higher than those in adjacent normal tissues (*P* < 0.001); however, no statistically significant difference was observed between grades 1, 2, and 3 (*P* > 0.05) (Figure 4E).

### 3.3 Relationship between RFC4 and the prognosis of patients with CC

**FIGURE 5:**
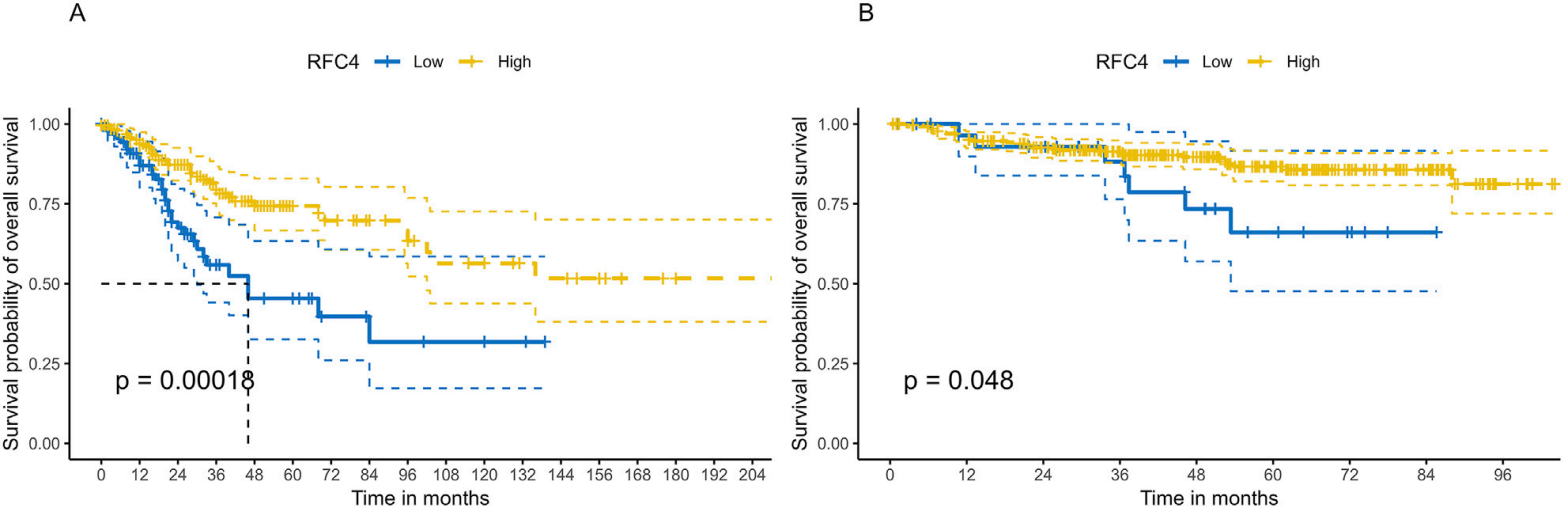
Kaplan–Meier survival analysis of the effect of RFC4 expression on survival in human CESC. **(A)** Overall survival using the TCGA database. **(B)** Validation of survival using the GSE44001 dataset.

**FIGURE 6:**
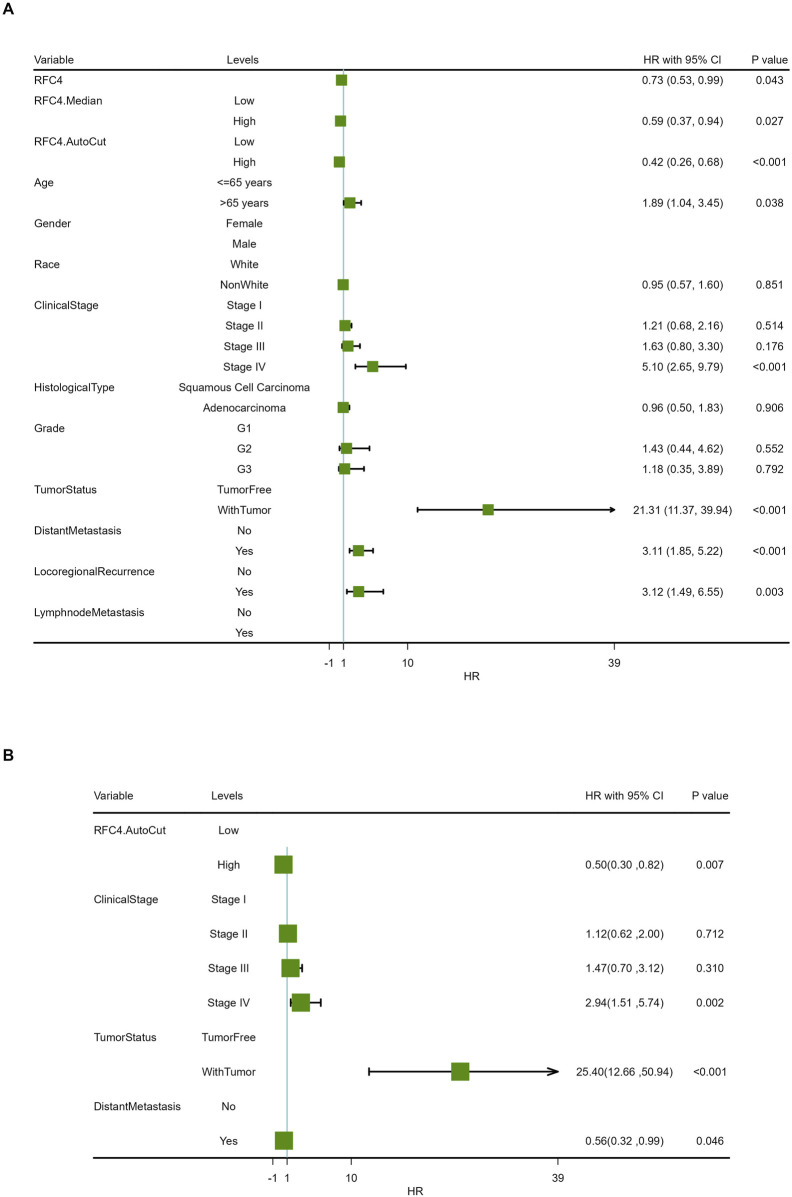
Forest map of the effect of RFC4 expression on overall survival in the Cox proportional-hazards model. Results of **(A)** univariate and **(B)** multivariate prognostic analysis via stepwise regression.

To explore the relationship between RFC4 and the prognosis of CC patients, we introduced the RFC4 expression level as a continuous variable into a Cox univariate regression model and simultaneously used the median value (RFC4.Median) and optimal threshold (RFC4.AutoCut) of RFC4 for analysis. The univariate regression results are showed in Table 1. RFC4 positively affected OS in the univariate Cox model, regardless of whether it was used as a continuous or categorical variable. Compared to RFC4 in the form of a continuous variable and median value, the best cutoff value had a higher effect value. Using the best cutoff value of 5.904, patients with CC in the TCGA database were divided into the RFC4^High^ (n = 211) and the RFC4^Low^ (n = 95) groups. Kaplan-Meier survival analysis showed that patients with high RFC4 expression had significantly better OS than those with low RFC4 expression (*P* < 0.001) (Figure 5A). The GSE44001 dataset was used to validate these results, and the difference was statistically significant (*P* = 0.048) (Figure 5B). Univariate analysis showed that RFC4, clinical stage, tumor status, distant metastasis, and locoregional recurrence were potential factors for the OS of patients with CC (*P* < 0.05), as shown in Table 1. A following multivariate COX regression analysis based on the above five positive variables was developed, and the results were presented in Table 2. In the stepwise regression multivariate model, locoregional recurrence was excluded because of strong collinearity. Finally, the results from the stepwise regression multivariate model showed that RFC4.AutoCut significantly affected the OS (HR = 0.50, 95%CI: 0.30-0.82, *P* = 0.007), which suggested that RFC4 has independent prognostic value in CC. The prognostic value of RFC4 is shown in Figures 6A,B.

**TABLE 1:** Univariate analysis of the prognostic ability of RFC4 in patients with CC.

Characteristics	Levels	Beta	Se	HR (95% CI for HR)	Statistics (Z value)	P
RFC4		−0.32	0.16	0.73 (0.53, 0.99)	−2.026	0.043
RFC4.Median	Low					
	High	−0.53	0.24	0.59 (0.37, 0.94)	−2.215	0.027
RFC4.AutoCut	Low					
	High	−0.86	0.24	0.42 (0.26, 0.68)	−3.585	<0.001
Age	≤65 years					
	>65 years	0.64	0.31	1.89 (1.04, 3.45)	2.077	0.038
Race	White					
	NonWhite	−0.05	0.26	0.95 (0.57, 1.60)	−0.188	0.851
Clinical stage	Stage I					
	Stage II	0.19	0.29	1.21 (0.68, 2.16)	0.652	0.514
	Stage III	0.49	0.36	1.63 (0.80, 3.30)	1.352	0.176
	Stage IV	1.63	0.33	5.10 (2.65, 9.79)	4.889	<0.001
Histological type	Squamous cell carcinoma					
	Adenocarcinoma	−0.04	0.33	0.96 (0.50, 1.83)	−0.119	0.906
Grade	G1					
	G2	0.36	0.60	1.43 (0.44, 4.62)	0.594	0.552
	G3	0.16	0.61	1.18 (0.35, 3.89)	0.264	0.792
Tumor status	Tumor free					
	With tumor	3.06	0.32	21.31 (11.37, 39.94)	9.548	<0.001
Distant metastasis	No					
	Yes	1.13	0.26	3.11 (1.85, 5.22)	4.278	<0.001
Locoregional recurrence	No					
	Yes	1.14	0.38	3.12 (1.49, 6.55)	3.009	0.003

**TABLE 2:** Multivariate analysis of prognostic ability of RFC4 in patients with CC.

Characteristics	Levels	Beta	Se	HR (95% CI for HR)	Statistics (Z value)	*P*
RFC4.AutoCut	Low					
	High	−0.70	0.26	0.50 (0.30,0.82)	2.719	0.007
Clinical stage	Stage I					
	Stage II	0.11	0.30	1.12 (0.62,2.00)	0.369	0.712
	Stage III	0.39	0.38	1.47 (0.70,3.12)	1.016	0.310
	Stage IV	1.08	0.34	2.94 (1.51,5.74)	3.166	0.002
Tumor status	Tumor free					
	With tumor	3.23	0.36	25.40 (12.66,50.94)	9.107	<0.001
Distant metastasis	No					
	Yes	−0.58	0.29	0.56 (0.32,0.99)	1.997	0.046

### 3.4 Analysis of DEGs and functional enrichment related to RFC4

**FIGURE 7:**
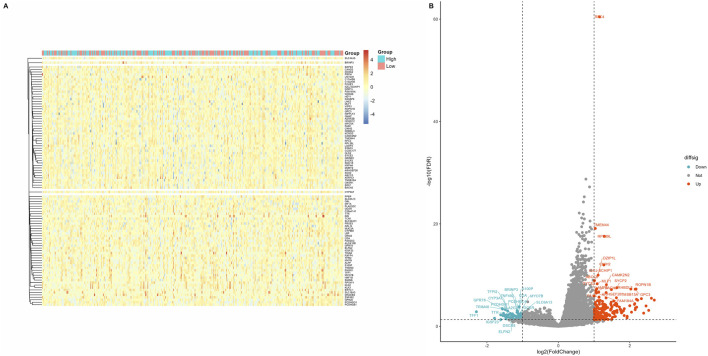
Screening of differentially expressed genes in different RFC4 statuses in the CESC cohort. **(A)** Heatmap showing 50 genes with the most significant upregulation and downregulation. **(B)** Volcano plot of differentially expressed genes.

Patients with CC in the TCGA database were divided into groups using the median RFC4 expression value, and differential expression analysis was conducted. Using |logs2 FC| values ≥1 and *P* < 0.05 as screening criteria, 291 DEGs were identified, of which 66 genes were overexpressed and 225 genes were underexpressed. Detailed information on the DEGs is provided in Supplementary Table S1. A heatmap and volcano map were created (Figures 7A,B).

**FIGURE 8:**
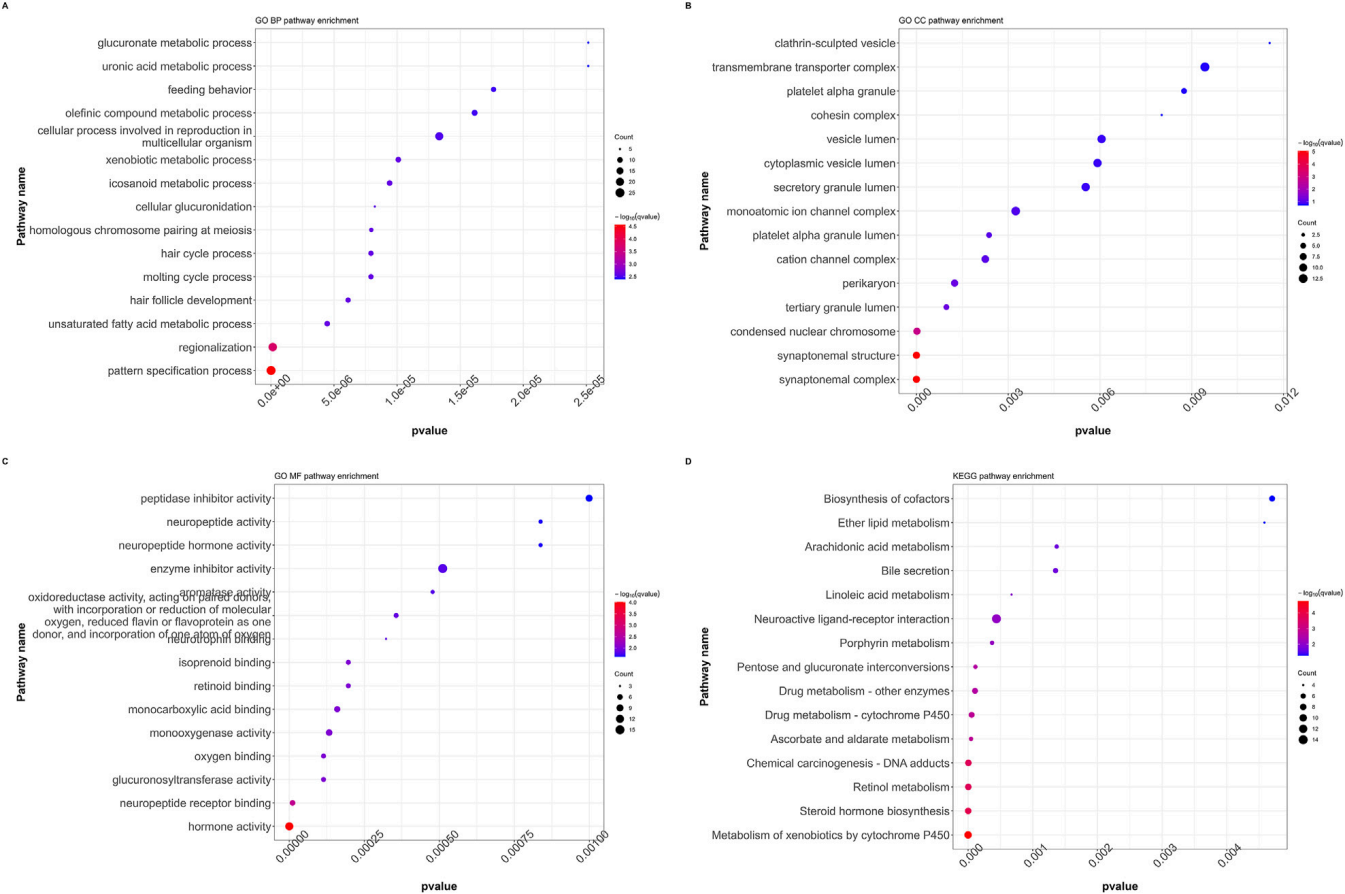
Functional annotation and pathway enrichment analysis of differentially expressed genes in the CESC cohort. GO functional annotation for **(A)** biological processes (BP), **(B)** cellular composition (CC), and **(C)** molecular functions (MF). **(D)** KEGG pathway enrichment analysis. (GO: Gene Ontology, KEGG: Kyoto Encyclopedia of Genes and Genomes, GSEA: Gene Set Enrichment Analysis).

All DEGs with significant transcriptional differences between the RFC4^High^ group and RFC4^Low^ group were screened and underwent functional enrichment analysis. The results showed that the biological processes were mainly enriched in pattern specification process, regionalization, unsaturated fatty acid metabolic process, hair follicle development, and molting cycle process. The cellular composition was mainly enriched in the synaptonemal complex, synaptonemal structure, condensed nuclear chromosome, tertiary granule lumen and perikaryon. The molecular functions were mainly enriched in hormone activity, neuropeptide receptor binding, glucuronosyltransferase activity, oxygen binding, monooxygenase activity and monocarboxylic acid binding. The KEGG pathways were mainly enriched in the metabolism of xenobiotics by cytochrome p450, steroid hormone biosynthesis, retinol metabolism, Chemical carcinogenesis - DNA adducts, and ascorbate and aldarate metabolism. Functional enrichment analysis indicated that RFC4 may play an important role in the occurrence and regulation of tumors. The functional enrichment analysis results are shown in Figures 8A–D. Detailed information on the functional enrichment analysis is provided in Supplementary Table S2.

### 3.5 Correlations between RFC4 and immunostimulators and immunoinhibitors

**FIGURE 9:**
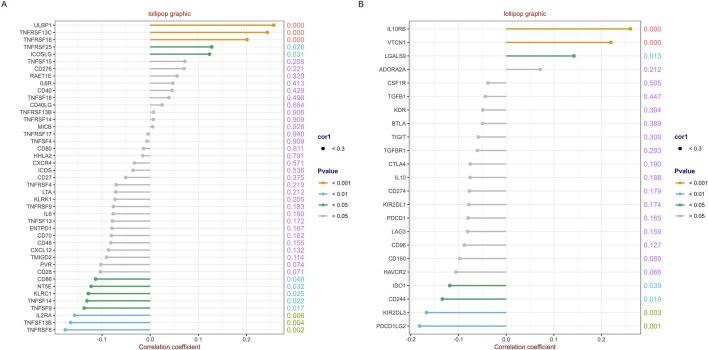
Correlation analysis between RFC4 and immunostimulators and immunoinhibitors. Correlation analysis of RFC4 and **(A)** 43 immunostimulators and **(B)** 23 immunoinhibitors.

A total of 43 immunostimulators were selected for correlation analysis. The detail results are shown in Supplementary Table S3 and Figure 9A. RFC4 was positively correlated with the expression of five immunostimulatory factors and negatively correlated with the expression of eight immunostimulatory factors. Similarly, 23 immunoinhibitors were selected for correlation analysis. The detail results were presented in Supplementary Table S4 and Figure 9B. RFC4 is positively correlated with the expression of three immunosuppressive factors and negatively correlated with the expression of four immunosuppressive factors.

### 3.6 Relationship between RFC4 and immune cell infiltration

**FIGURE 10:**
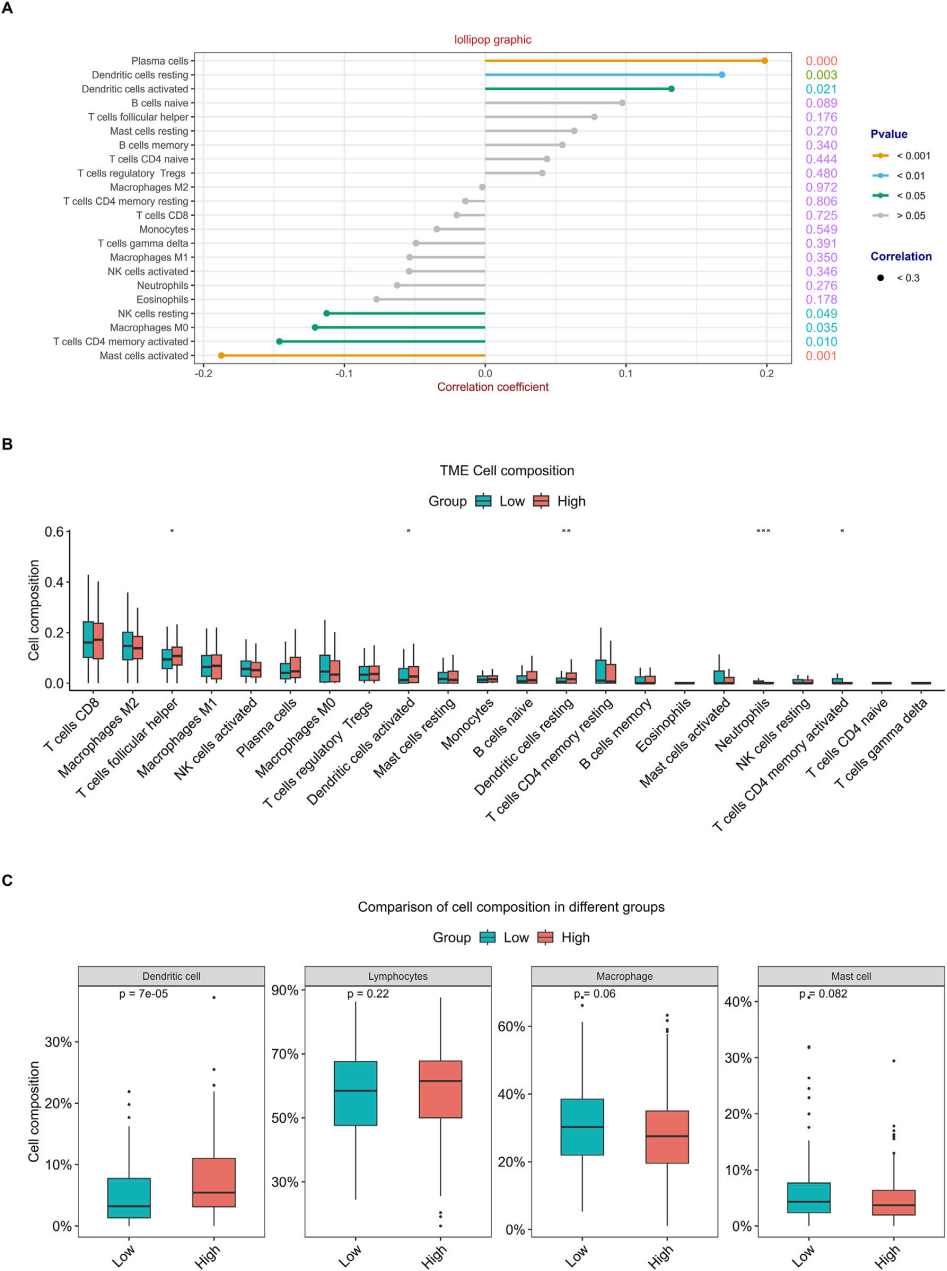
Analysis of tumor-infiltrating immune cell changes in different RFC4 statuses in the CESC cohort using CIBERSORT. **(A)** Correlation analysis of 22 immune cell types and RFC4. **(B)** Differential analysis of immune cell infiltration between the RFC4^High^ and RFC4^Low^ groups. **(C)** Four categories of tumor-infiltrating immune cells between the RFC4^High^ and RFC4^Low^ groups.

**FIGURE 11:**
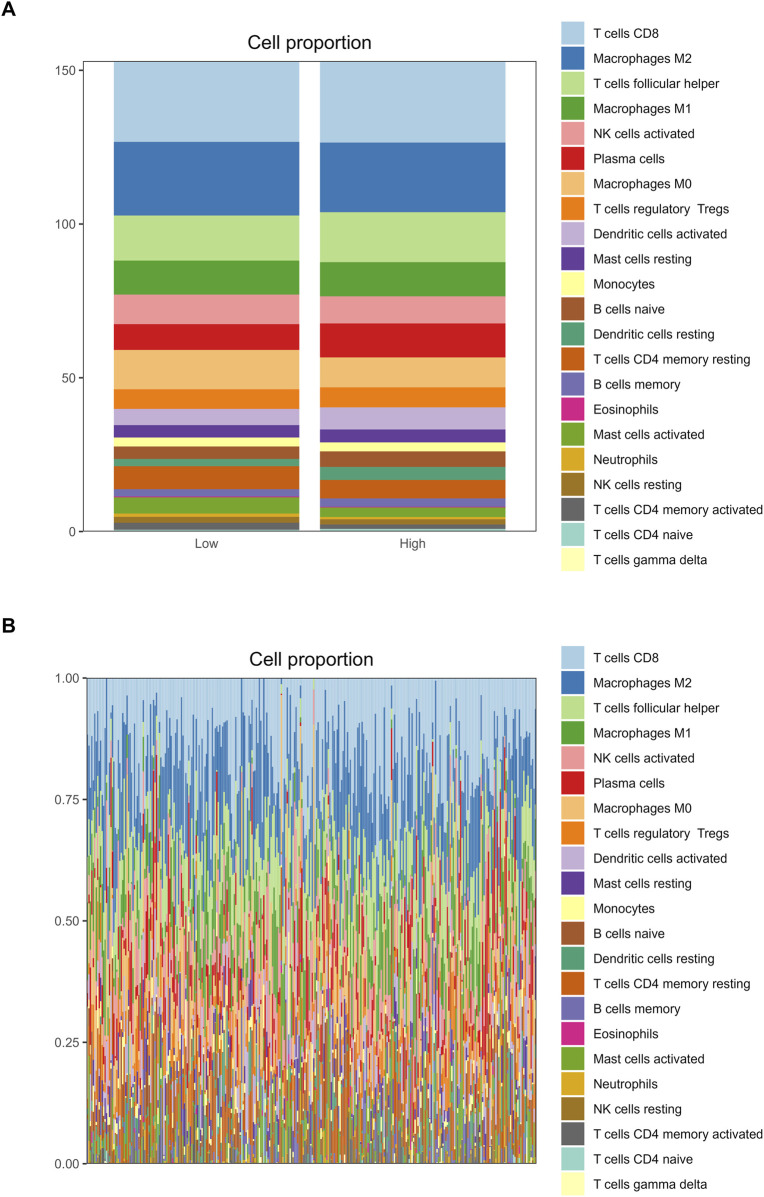
Analysis of tumor-infiltrating immune cell changes in different RFC4 statuses in the CESC cohort using CIBERSORT. **(A)** Proportion of 22 immune cell types between **(A)** the RFC4^High^ and RFC4^Low^ groups and **(B)** in all TCGA cervical cancer samples.

The correlation analysis of the 22 immune cell types and RFC4 gene expression levels from CIBERSORT is shown in Figure 10A and Supplementary Table S5. Among them, activated mast cells, activated CD4 memory T cells, M0 macrophages and resting natural killer (NK) cells weresignificantly negatively correlated with RFC4, whereas activated dendritic cells, resting dendritic cells, and plasma cells were significantly positively correlated with RFC4. The infiltration of 22 types of immune cells in the RFC4^High^ and RFC4^Low^ groups is shown in Figure 10B. With median expression value of RFC4, our results showed that follicular helper T cells, activated dendritic cells and resting dendritic cells showed relatively more infiltration in the RFC4^High^ group, while neutrophils and activated CD4 memory T cells had relatively more infiltration in the RFC4^Low^ group. Furthermore, we regrouped immune cells into four categories and found that dendritic cells showed the most significant differences between the RFC4^High^ and RFC4^Low^ groups, as shown in Figure 10C. The immune cell infiltration in different RFC4 groups and CC samples is shown in Figures 11A,B.

**FIGURE 12:**
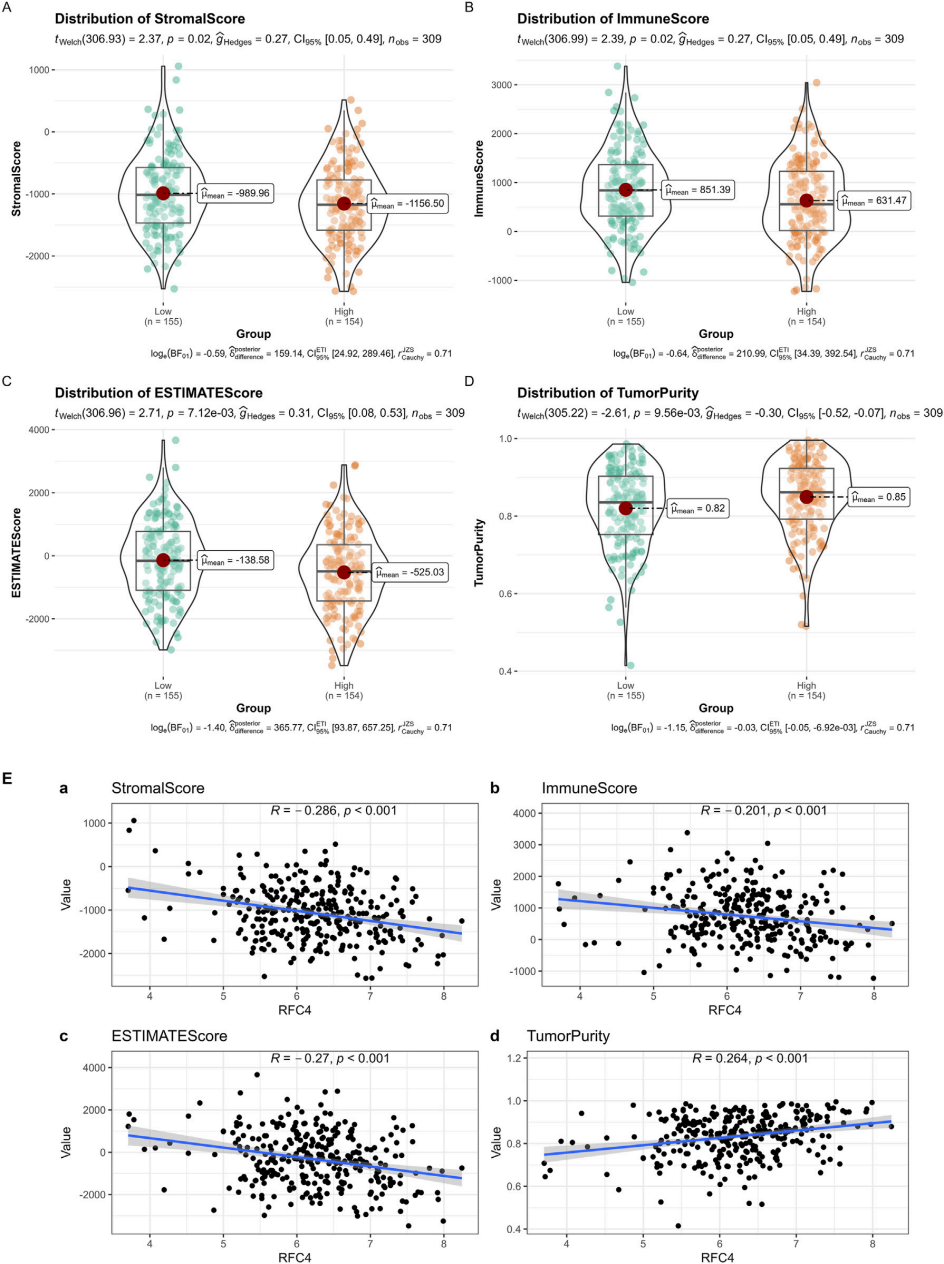
Analysis of tumor-infiltrating immune cell changes between the RFC4High and RFC4Low groups in the CESC cohort using ESTIMATE. **(A)** Stromal scores, **(B)** immune scores, **(C)** ESTIMATE scores, and **(D)** tumor purity. **(E)** Correlation analysis between RFC4 and (a) stromal scores, (b) immune scores, (c) ESTIMATE scores, and (d) tumor purity.

The results of the ESTIMATE showed that cancer tissues in the RFC4^High^ group had lower stromal, immune, and ESTIMATE scores and higher tumor purity, as shown in Figures 12A–D. The correlation analysis between RFC4 expression levels and stromal scores, immune scores, ESTIMATE scores, and tumor purity from ESTIMATE are shown in Figure 12E. Among them, activated mast cells and stromal, immune and ESTIMATE scores were significantly negatively correlated with RFC4, whereas tumor purity was positively correlated with RFC4 expression.

### 3.7 Expression of RFC4 protein was significantly increased in CC compared with normal tissues

**FIGURE 13:**
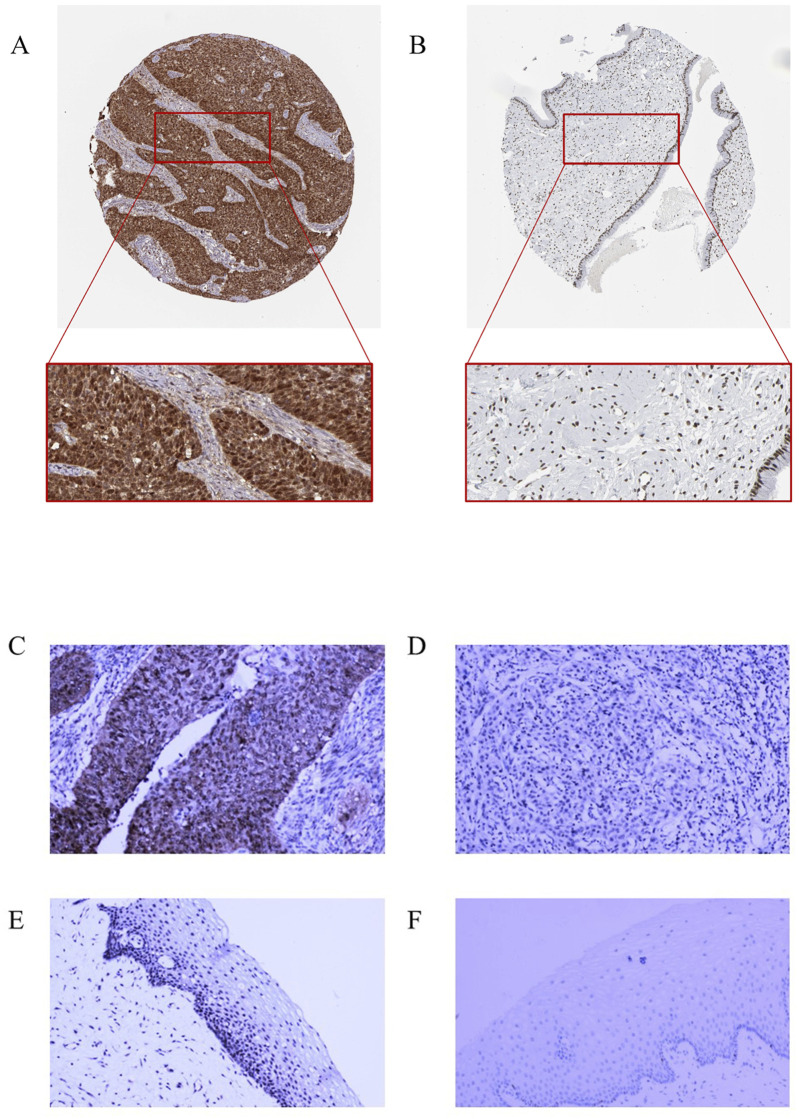
Immunohistochemical staining for RFC4. **(A)** High RFC4 expression in CESC cancer tissues from the HPA database. **(B)** Low RFC expression in normal tissues from the HPA database. **(C)** Strong positive expression of RFC4 in CESC cancer tissues from the clinical set. **(D)** Weak positive expression of RFC4 in cancer tissues from the clinical set. **(E)** Moderately positive expression of RFC4 in normal CESC tissues from the clinical set. **(F)** Negative RFC4 expression in normal tissues from the clinical set.

To verify RFC4 protein expression in CC tissues, we analyzed IHC staining images from the HPA database. The results showed that RFC4 protein exhibited moderate-to-strong expression in 28 CC tissues samples, and only one CC tissue sample showed weak expression. IHC staining of RFC4 protein in CC and normal cervical tissues from the HPA database is shown in Figures 13A,B. The IHC staining results extracted from our real-world cohort were consistent with those obtained from the HPA database. The rate of moderate-to-strong expression of RCF4 in CC tissues was 86.67% (13/15), while that in adjacent tissues was 46.67% (7/15), and the difference was statistically significant (χ^2^ = 5.412, *P* = 0.02), as shown in Figures 13C–F.

## 4 Discussion

This study combined a real-world cohort and publicly available databases and demonstrated that high RFC4 expression is associated with the occurrence of CC and better prognosis. Further research has found that abnormal RFC4 expression may regulate the tumor microenvironment by altering the status of infiltrating immune cells within the tumor tissues.

Persistent high-risk HPV infection is an important pathogenic factor for CC (Tao et al., 2015); however, only a small percentage of infected women developed CC, and not all patients with CC are infected with HPV (Hu and Ma, 2018; Perkins et al., 2023). Therefore, HPV infection may not be the only factor that induces CC, which increases the difficulty of CC screening (Bedell et al., 2020; Güzel et al., 2021). The occurrence of tumors is highly correlated with the abnormal expression of multiple genes, which may have value in the diagnosis of cancer. Diagnostic models based on abnormal gene expression have also been developed for CC Liao et al., 2023). Therefore, it is crucial to explore the genes that are differentially expressed between CC and normal cervical tissues, to study the genetic pathogenesis of CC.

The RFC family is a complex composed of RFC1, RFC2, RFC3, RFC4, and RFC5 subunits and is a primer recognition factor for DNA polymerases (Guan et al., 2023). RFC and RFC-like complexes (RLCs) mediate chromatin engagement of PCNA, which was first purified from human CC HeLa cells and contains five subunits of different sizes (Kang et al., 2019; Chen et al., 2023). RLCs can selectively bind to the ends of DNA primers and act as primer recognition factors for DNA polymerase to participate in DNA replication (Sisakova et al., 2017). RLCs not only increases the affinity between DNA polymerase and primer ends but also reduce the amount of PCNA required to activate DNA polymerase (Liu et al., 2022). The RFC complex exhibits DNA-dependent ATPase activity, which is necessary for activating DNA polymerase (Liu et al., 2022). Research has shown that the RFC complex contains a 5'DNA binding site responsible for transferring the 9-1-1 heterotrimeric clamp onto DNA, playing a role in DNA break repair (Li et al., 2022).

The role of RFC complexes in cancer occurrence and progression has received increasing attention (Yu et al., 2024; Bermúdez-Guzmán, 2021). RFCs exhibit biological activity in various malignant tumors and may play important roles in the proliferation, progression, invasion, and metastasis of cancer cells (Yang et al., 2019; Wang et al., 2019). Based on the cellular and histological characteristics of the tumors, they can serve as oncogenes or tumor suppressors. RFC4 is a regulatory protein primarily present in the nucleus, with a relative molecular weight of 37,000 Da (Maga et al., 1997). RFC4 exists in the RFC complex of DNA and participates in the formation of DNA replication complexes, thus initiating DNA replication. RFC4 is also involved in various important cellular processes, including DNA strand extension, DNA repair, phosphoinositol-related signaling pathways (Krause et al., 2001). Previous studies have shown that RFC4 is required for cancer cell proliferation and may play a pivotal role in tumorigenesis in most cancer (Guan et al., 2023; Yang et al., 2019; Wang et al., 2019). In a previous study, whole-genome RNAi screening showed that RFC4 mediates radiotherapy tolerance in colorectal cancer by promoting repair of non-homologous end connections (Wang et al., 2019). The expression level of RFC4 has also been associated with the progression of colorectal cancer and can be used for prognosis prediction of colorectal cancer (Wang et al., 2019). Bioinformatics analysis has confirmed that RFC4 is a potential therapeutic target for liver cancer (Yang et al., 2019). However, the potential effect of RFC4 on CC remains unclear.

In the present study, we first explored the expression of RFC4 in multiple cancers using a pan-cancer analysis. Consistent with previous reports, RFC4 was highly expressed in various cancers, indicating its involvement in the tumorigenesis. Compared to adjacent tissues, RFC4 was highly expressed in CC tissues. However, further research found no significant correlation between RFC4 and clinical staging of CC, and whether RFC4 is involved in CC progression is still unclear. Based on clinical characteristics such as age and tumor differentiation, we found that high RFC4 expression was stable in different subgroups of patients with CC, indicating that differential expression of RFC4 is reliable.

Based on the differential expression of RFC4 in patients with CC, RFC4 appears to be an oncogenic gene. Surprisingly, however, we found that high RFC4 expression is a sign of better prognosis in CC. We speculate that the body may activate immune responses through various anti-cancer mechanisms to exert anti-cancer effects after tumor occurrence and that RCF4 may be involved in these processes; thus, its expression is further enhanced. The contradiction between the prognostic and diagnostic value of RFC4 suggests the complexity of its role, and future studies should continue identifying the biological functions of RFC4. Zhang et al. determined the potential role of RFC4 in cervical carcinogenesis through comprehensive study of gene expression profiles; and RFC4 was proposed as a novel alternative biomarker to determine HSIL and HSIL+, as well as an independent prognostic biomarker for cervical squamous cell carcinoma (Zhang et al., 2022). Our results are consistent with those of Zhang et al. (2022). Different from the research focus of Zhang et al., we have provided the effect of RFC4 on tumor immune microenvironment.

To reveal the biological role of RFC4 in CC, we conducted GO analysis and KEGG functional enrichment analyses. RFC4 was associated with cellular functions such as DNA replication, cell cycle, mismatch repair, base excision repair, and nucleotide excision repair. We speculate that overactivation of DNA repair mechanisms may be the reason for the high survival rate of patients with CC with high RFC4 expression.

The tumor immune microenvironment (TIME) is involved in tumor clonal evolution, growth, metastasis, prognosis, drug resistance, and treatment outcomes (Deng et al., 2023). Therefore, we used CIBERSORT and ESTIMATE to analyze the relationship between RFC4 expression and immune cell infiltration determine the immune characteristics of RFC4 in CC. Firstly, we evaluated the correlations between RFC4 and immunostimulators and immunoinhibitors. With respect to immune-stimulating factors, we found that RFC4 was positively correlated with the expression of ULBP1, TNFRSF13C, TNFRSF18, TNFRSF25 and ICOSLG, and negatively correlated with the expression of CD86, NT5E, KLRC1, TNFSF14, IL2RA, TNSF13B, and TNFRSF8. From the perspective of immunosuppressive factors, we found that RFC4 was positively correlated with the expression of IL10RB, VTCN1 and LGALS9, and negatively correlated with the expression of IDO1, CD244, KIR2DL3, and PDCD1LG2. Therefore, RFC4 seems more closely related to immunostimulation, which partly explains why high RFC4 expression is associated with a better CC prognosis. Second, we evaluated the relationship between RFC4 expression and immune cell infiltration. We found that activated mast cells, activated CD4 memory T cells, M0 macrophages, and resting natural killer (NK) cells were significantly negatively correlated with RFC4, whereas activated dendritic cells, resting dendritic cells, and plasma cells were significantly positively correlated with RFC4. Numerous studies have shown that mast cells, CD4^+^ memory T cells, M0 macrophages, and quiescent natural killer (NK) cells are associated with cancer recurrence and progression, whereas dendritic cells, CD8^+^ T cells, and M1 macrophages may be effector cells for anticancer treatment (Mao et al., 2024; He et al., 2022; Leone and Powell, 2020). Overall, the expression of RCF4 in CC was correlated with positive anti-cancer cells, which further explains why high RFC4 expression is an indicator of good prognosis. Third, our results showed that RFC4 expression was positively correlated with tumor purity, and negatively correlated with stromal, immune and ESTIMATE scores. Overall, RFC4 acts as a positive anti-cancer gene, suggesting that the development of immunotherapy based on RFC4 may have little effect, which can help in planning research. Finally, our study used the HPA database to validate the expression of RFC4 protein in CC, and the results were consistent with the expression of RFC4 mRNA.

Our study had some limitations. First, our study analyzed only the TCGA, GTEx, and GEO databases, and the results should be validated in clinical cohorts in the future. Second, the biological function of RFC4 protein expression in CC cells should be experimentally validated. Furthermore, the factors and upstream and downstream signaling pathways that regulate RFC4 *in vivo* should be explored. Finally, some future research ideas on RFC4 need to be mentioned. The immune infiltration analysis of RFC4 in our present study only relies on CIBERSORT and ESTIMATE; thus, it is necessary to perform IHC staining or multiplex immunofluorescence to confirm the spatial relationship between RFC4 and specific immune cells (e.g., T cells, mast cells) to verify the results from bioinformatics tools. In addition, we need to further conduct RFC4 function inhibition experiments to determine the effect of knocking down *RFC4* on the malignant behavior of CC cells.

## 5 Conclusion

In conclusion, this study elucidates the relationship between high RFC4 expression and the occurrence, malignant phenotype, and prognosis of CC. In addition, our study revealed the mechanism by which RFC4 exerts antitumor effects by shaping the immunostimulatory tumor microenvironment, including immunostimulatory and dendritic cell infiltration. Overall, the biological role of RFC4 is complex, mainly manifested in its potential to promote cancer development in the early stages. However, in cancer progression, it exerts anti-cancer effects through immunostimulation.

## Data Availability

The original contributions presented in the study are included in the article/Supplementary Material, further inquiries can be directed to the corresponding author.

## Glossary

GEO: Gene expression omnibus
TCGA: The cancer genome atlas
DEGs: differentially expressed genes
GO: Gene Ontology
KEGG: Kyoto Encyclopedia of Genes and Genomes
TME: Tumor microenvironment
OS: Overall survival
ACC: Adrenocortical Carcinoma
BLCA: Bladder Urothelial Carcinoma
BRCA: Breast invasive carcinoma
CESC: Cervical squamous cell carcinoma and endocervical adenocarcinoma
CHOL: Cholangio carcinoma
COAD: Colon adenocarcinoma
DLBC: Lymphoid Neoplasm Diffuse Large B-cell Lymphoma
ESCA: Esophageal carcinoma
GBM: Glioblastoma multiforme
HNSC: Head and Neck squamous cell carcinoma
KICH: Kidney Chromophobe
KIRC: Kidney renal clear cell carcinoma
KIRP: Kidney renal papillary cell carcinoma
LAML: Acute Myeloid Leukemia
LGG: Brain Lower Grade Glioma
LIHC: Liver hepatocellular carcinoma
LUAD: Lung adenocarcinoma
LUSC: Lung squamous cell carcinoma
MESO: Mesothelioma
OV: Ovarian serous cystadenocarcinoma
PAAD: Pancreatic adenocarcinoma
PCPG: Pheochromocytoma and Paraganglioma
PRAD: Prostate adenocarcinoma
READ: Rectum adenocarcinoma
SARC: Sarcoma
SKCM: Skin Cutaneous Melanoma
STAD: Stomach adenocarcinoma
TGCT: Testicular Germ Cell Tumors
THCA: Thyroid carcinoma
THYM: Thymoma
UCEC: Uterine Corpus Endometrial Carcinoma
UCS: Uterine Carcinosarcoma
UVM: Uveal Melanoma
